# Novel Therapies for Hepatocellular Carcinoma

**DOI:** 10.3390/cancers12103049

**Published:** 2020-10-20

**Authors:** Lorenza Rimassa

**Affiliations:** 1 Medical Oncology and Hematology Unit, Humanitas Cancer Center, Humanitas Clinical and Research Center-IRCCS, 20089 Rozzano (Milan), Italy; lorenza.rimassa@hunimed.eu; 2 Department of Biomedical Sciences, Humanitas University, 20090 Pieve Emanuele (Milan), Italy

Since 2007, for patients with advanced- or intermediate-stage hepatocellular carcinoma (HCC) unsuitable for locoregional treatments and with preserved liver function, the multikinase inhibitor (MKI) sorafenib has been the worldwide standard of care [1,2]. After ten years of unsatisfactory results, additional agents with prevalent antiangiogenic activity have been approved based on positive phase 3 data: lenvatinib [3] in the first-line setting, regorafenib [4], cabozantinib [5], and ramucirumab [6] for patients previously treated with sorafenib.

Also, immune checkpoint inhibitors (ICI) targeting the programmed cell death receptor-1 (PD-1) as monotherapy or in combination with a cytotoxic T-lymphocyte antigen 4 (CTLA-4)-blocking antibody have been granted accelerated approval in sorafenib-pretreated patients based on phase 1/2 data [7,8,9]. Although phase 3 trials testing ICI alone as first- and second-line therapy failed to meet their primary endpoints [10,11], based on the potential interplay between antiangiogenic drugs and immunotherapy, novel combinations of ICI plus antiangiogenics have been tested in untreated patients with encouraging preliminary results [12,13]. Moreover, other important early phase studies have tested molecular therapies directed against different novel targets, such as transforming growth factor-beta, MET (the hepatocyte growth factor receptor), and fibroblast growth factor receptor 4 [14].

Recently, the phase 3 IMbrave150 trial demonstrated the superiority of the combination of atezolizumab (a monoclonal antibody blocking the programmed cell death-ligand 1 [PD-L1]) plus bevacizumab (a monoclonal antibody against the vascular endothelial growth factor [VEGF]) compared to sorafenib in the front-line setting and established the new standard of care for these patients [15].

Further combinations of anti-PD-1/PD-L1 and MKI or anti-CTLA-4 and are being evaluated in phase 3 trials and might expand the therapeutic scenario in the next years [14,16,17,18].

However, an important unmet need is currently represented by the lack of clinical and/or biological factors and/or biomarkers that can guide therapeutic choices, apart from AFP, which is used to select patients for ramucirumab. This unmet need is being addressed in several studies that integrate translational research with the aim of better defining the biological tumor profile and identifying tumor and blood biomarkers to select patients who may really benefit from a specific therapy [14].

In this rapidly evolving scenario, it is extremely important that physicians are updated and aware of novel therapeutic options in order to make the best use of them in various clinical settings [19,20].

This Special Issue will highlight the key open issues and future perspectives for patients with advanced HCC, such as novel therapies and approaches, novel therapeutic targets, and biomarkers.

## References

[1] Llovet J.M., Ricci S., Mazzaferro V., Hilgard P., Gane E., Blanc J.F., De Oliveira A.C., Santoro A., Raoul J.-L., Forner A. (2008). Sorafenib in advanced hepatocellular carcinoma. N. Engl. J. Med..

[2] Cheng A.L., Kang Y.K., Chen Z., Tsao C.-J., Qin S., Kim J.S., Luo R., Feng J., Ye S., Yang T.-S. (2009). Efficacy and safety of sorafenib in patients in the Asia-Pacific region with advanced hepatocellular carcinoma: A phase III randomised, double-blind, placebo-controlled trial. Lancet Oncol..

[3] Kudo M., Finn R.S., Qin S., Han K.H., Ikeda K., Piscaglia F., Baron A., Park J.-W., Han G., Jassem J. (2018). Lenvatinib versus sorafenib in first-line treatment of patients with unresectable hepatocellular carcinoma: A randomised phase 3 non-inferiority trial. Lancet.

[4] Bruix J., Qin S., Merle P., Granito A., Huang Y.H., Bodoky G., Pracht M., Yokosuka O., Rosmorduc O., Breder V. (2017). Regorafenib for patients with hepatocellular carcinoma who progressed on sorafenib treatment (RESORCE): A randomised, double-blind, placebo-controlled, phase 3 trial. Lancet.

[5] Abou-Alfa G.K., Meyer T., Cheng A.L., El-Khoueiry A.B., Rimassa L., Ryoo B.Y., Cicin I., Merle P., Chen Y., Park J.-W. (2018). Cabozantinib in patients with advanced and progressing hepatocellular carcinoma. N. Engl. J. Med..

[6] Zhu A.X., Kang Y.K., Yen C.J., Finn R.S., Galle P.R., Llovet J.M., Assenat E., Brandi G., Pracht M., Lim H.Y. (2019). Ramucirumab after sorafenib in patients with advanced hepatocellular carcinoma and increased α-fetoprotein concentrations (REACH-2): A randomised, double-blind, placebo-controlled, phase 3 trial. Lancet Oncol..

[7] El-Khoueiry A.B., Sangro B., Yau T., Crocenzi T.S., Kudo M., Hsu C., Kim T.-Y., Choo S.-P., Trojan J., Welling T.H. (2017). Nivolumab in patients with advanced hepatocellular carcinoma (CheckMate 040): An open-label, non-comparative, phase 1/2 dose escalation and expansion trial. Lancet.

[8] Zhu A.X., Finn R.S., Edeline J., Cattan S., Ogasawara S., Palmer D., Verslype C., Zagonel V., Fartoux L., Vogel A. (2018). Pembrolizumab in patients with advanced hepatocellular carcinoma previously treated with sorafenib (KEYNOTE-224): A non-randomised, open-label phase 2 trial. Lancet Oncol..

[9] Yau T., Kang Y.K., Kim T.Y., El-Khoueiry A.B., Santoro A., Sangro B., Melero I., Kudo M., Hou M.M., Matilla A. (2020). Efficacy and safety of nivolumab plus ipilimumab in patients with advanced hepatocellular carcinoma previously treated with sorafenib: The CheckMate 040 randomized clinical trial. JAMA Oncol..

[10] Yau T., Park J.W., Finn R.S., Cheng A., Mathurin P., Edeline J., Kudo M., Han K.-H., Harding J., Merle P. (2019). LBA38_PR: CheckMate 459: A randomized, multi-center phase 3 study of nivolumab (NIVO) vs sorafenib (SOR) as first-line (1L) treatment in patients (PTS) with advanced hepatocellular carcinoma (AHCC). Ann. Oncol..

[11] Finn R.S., Ryoo B.Y., Merle P., Kudo M., Bouattour M., Lim H.Y., Breder V., Edeline J., Chao Y., Ogasawara S. (2020). Pembrolizumab as second-line therapy in patients with advanced hepatocellular carcinoma in KEYNOTE-240: A randomized, double-blind, phase III trial. J. Clin. Oncol..

[12] Lee M.S., Ryoo B.Y., Hsu C.H., Numata K., Stein S., Verret W., Hack S.P., Spahn J., Liu B., Abdullah H. (2020). Atezolizumab with or without bevacizumab in unresectable hepatocellular carcinoma (GO30140): An open-label, multicentre, phase 1b study. Lancet Oncol..

[13] Finn R.S., Ikeda M., Zhu A.X., Sung M.W., Baron A.D., Kudo M., Okusaka T., Kobayashi M., Kumada H., Kaneko S. (2020). Phase Ib study of lenvatinib plus pembrolizumab in patients with unresectable hepatocellular carcinoma. J. Clin. Oncol..

[14] Faivre S., Rimassa L., Finn R.S. (2020). Molecular therapies for HCC: Looking outside the box. J. Hepatol..

[15] Finn R.S., Qin S., Ikeda M., Galle P.R., Ducreux M., Kim T.Y., Kudo M., Breder V., Merle P., Kaseb A.O. (2020). Atezolizumab plus bevacizumab in unresectable hepatocellular carcinoma. N. Engl. J. Med..

[16] Kelley R.K., Sangro B., Harris W.P., Ikeda M., Okusaka T., Kang Y.-K., Qin S., Tai W.M.D., Lim H.Y., Yau T. (2020). Efficacy, tolerability, and biologic activity of a novel regimen of tremelimumab (T) in combination with durvalumab (D) for patients (pts) with advanced hepatocellular carcinoma (aHCC). J. Clin. Oncol..

[17] Kelley R.K., WOliver J., Hazra S., Benzaghou F., Yau T., Cheng A.L., Rimassa L. (2020). Cabozantinib in combination with atezolizumab versus sorafenib in treatment-naive advanced hepatocellular carcinoma: COSMIC-312 Phase III study design. Future Oncol..

[18] Cheng A.-L., Hsu C., Chan S.L., Choo S.-P., Kudo M. (2020). Challenges of combination therapy with immune checkpoint inhibitors for hepatocellular carcinoma. J. Hepatol..

[19] Rimassa L., Wörns M.A. (2020). Navigating the new landscape of second-line treatment in advanced hepatocellular carcinoma. Liver Int..

[20] Personeni N., Pressiani T., Rimassa L. (2020). Which choice of therapy when many are available? Current systemic therapies for advanced hepatocellular carcinoma. Health Sci. Rep..

